# Recent Origin of the Methacrylate Redox System in *Geobacter sulfurreducens* AM-1 through Horizontal Gene Transfer

**DOI:** 10.1371/journal.pone.0125888

**Published:** 2015-05-11

**Authors:** Oksana V. Arkhipova, Margarita V. Meer, Galina V. Mikoulinskaia, Marina V. Zakharova, Alexander S. Galushko, Vasilii K. Akimenko, Fyodor A. Kondrashov

**Affiliations:** 1 Scryabin’s Institute of Biochemistry and Physiology of Microorganisms, Pushchino 142290, Russia; 2 Bioinformatics and Genomics Programme, Centre for Genomic Regulation (CRG) 88 Dr. Aiguader, 08003 Barcelona, Spain; 3 Universitat Pompeu Fabra (UPF), 08003 Barcelona, Spain; 4 Branch of Shemyakin & Ovchinnikov’s Institute of Bioorganic Chemistry, Pushchino 142290, Russia; 5 Agrophysical Research Institute RAS, Saint-Petersburg 195220, Russia; 6 Tomsk State University, Tomsk, 634050, Russia; 7 Institució Catalana de Recerca i Estudis Avançats (ICREA), 23 Pg. Lluís Companys, 08010 Barcelona, Spain; University of Alberta, CANADA

## Abstract

The origin and evolution of novel biochemical functions remains one of the key questions in molecular evolution. We study recently emerged methacrylate reductase function that is thought to have emerged in the last century and reported in *Geobacter sulfurreducens* strain AM-1. We report the sequence and study the evolution of the operon coding for the flavin-containing methacrylate reductase (Mrd) and tetraheme cytochrome *с* (Mcc) in the genome of *G*. *sulfurreducens* AM-1. Different types of signal peptides in functionally interlinked proteins Mrd and Mcc suggest a possible complex mechanism of biogenesis for chromoproteids of the methacrylate redox system. The homologs of the Mrd and Mcc sequence found in *δ-Proteobacteria* and *Deferribacteres* are also organized into an operon and their phylogenetic distribution suggested that these two genes tend to be horizontally transferred together. Specifically, the *mrd* and *mcc* genes from *G*. *sulfurreducens* AM-1 are not monophyletic with any of the homologs found in other *Geobacter* genomes. The acquisition of methacrylate reductase function by *G*. *sulfurreducens* AM-1 appears linked to a horizontal gene transfer event. However, the new function of the products of *mrd* and *mcc* may have evolved either prior or subsequent to their acquisition by *G*. *sulfurreducens* AM-1.

## Introduction

Anaerobic bacteria frequently use unsaturated organic compounds as terminal electron acceptors [1]. Among such forms of respiration, fumarate respiration of anaerobes has been studied most extensively [2–8]. During fumarate respiration bacterial cells reduce fumarate in the cytosol (e.g. *Wolinella succinogenes* and *Escherichia coli*) or in the periplasm (as in *Shewanella*). The cytosolic fumarate-reducing enzyme complex is located at the inner side of the cytoplasmic membrane and consists of 3 or 4 protein subunits [2–7]. Periplasmic fumarate reductases of the bacterial genus *Shewanella* are soluble monomers belonging to the flavocytochrome *c* family [9–16]. Data on enzyme systems and electron transport chain components that use other double-bond compounds as terminal electron acceptors are often fragmentary and contradictory or completely absent [1].

Anaerobic bacterium *Geobacter sulfurreducens* AM-1 was isolated in the study of decomposition of methacrylate industry waste [17]. The *G*. *sulfurreducens* AM-1 strain is capable of complete oxidation of acetate coupled to reduction of methacrylate (2-methylpropenoate), an anthropogenic compound that serves as the terminal acceptor of the bacterial reductase chain [18]. The study of *Geobacter* species (*Deltaproteobacteria*) is of applied interest due to their significant role in bioremediation of radioactive metals [19–22]. They serve as important agents in the global cycles of metals and carbon, reducing Fe(III) to Fe(II) and U(VI) to U(IV), oxidizing acetate and other organic compounds and participating in humus decomposition. Furthermore, they are fumarate-respiring organisms [19–21,23] and electrotrophs [24].

Transformation of methacrylate to isobutyrate occurs in the periplasm of bacterium *G*. *sulfurreducens* АМ-1 [25] by the periplasmic flavin-containing methacrylate reductase Mrd (50 kDa) [1,18]. Mrd activity depends on periplasmic tetraheme cytochrome *c* Mcc (30 kDa), which is the physiological electron donor for this enzyme. Furthermore, the two-component methacrylate redox system catalyzes reduction of acrylate, which is a compound found in nature [26], at a rate comparable to that for synthetic methacrylate, while lacking fumarate reduction [18].

Membranes of bacterium *G*. *sulfurreducens* АМ-1 contain menaquinone-8 (menaquinone with 8 isoprene residues in the side chain), which transfers reducing equivalents to the methacrylate redox system from the citric acid cycle [1,18]. The electron carrier from menaquinone to Mcc remains unknown, although the periplasmic cytochromes *c* (12.5 and 15.5 kDa) and the membrane cytochrome *c* (67.6 kDa) are possible candidates [27].

N-terminal amino acid sequences, 27 and 29 amino acids in length, respectively, were identified from purified Mrd and Mcc [18]. Previous analysis suggested that the Mrd sequence was homologous to flavocytochromes *c* in several bacterial and a few archaeal genomes [28]. However, the length of the Mrd fragment was not long enough to perform a comprehensive sequence analysis of the two proteins that have recently evolved into the methacrylate redox system. Furthermore, the Mcc amino acid sequence has not been investigated.

Methacrylate, a common monomer in polymer plastics and resins, is strictly a man-made molecule [29]. It is also the main substrate of the methacrylate redox system and, therefore, methacrylate-based respiration might have evolved sometime in the second half of the 20^th^ century. The *G*. *sulfurreducens* AM-1 strain is the only one known strain capable of methacrylate respiration [1,17] and, therefore, the sequences of the methacrylate redox system genes provide an unparalleled opportunity to study the evolutionary history of a novel system of respiration.

Here we report the sequence of the two genes of the methacrylate redox system from the *G*. *sulfurreducens* АМ-1 genome, analyze their translation products and study their evolutionary origins.

## Results

### Organization of the mrd and mcc genes in the Geobacter sulfurreducens AM-1 genome

**Fig 1:**
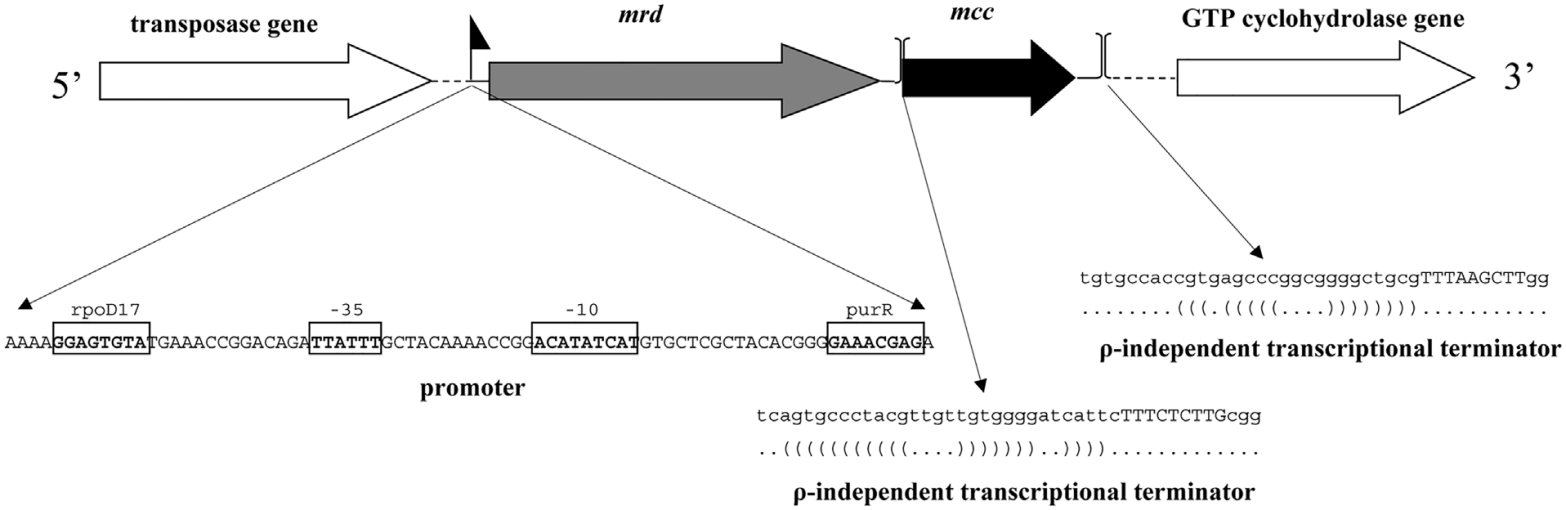
Organization of the operon of the methacrylate redox system in *Geobacter sulfurreducens* AM-1. The putative promoter is marked as small flag, ρ-independent transcriptional terminators are marked by parentheses. Conservative sequences of promoter -35, -10 and sites of the binding of transcriptional factors are enclosed in frames.

We sequenced the genome of *G*. *sulfurreducens* AM-1, obtaining a draft with a single contig. To localize the *mrd* and *mcc* genes, we mapped the previously identified short 27 and 29 amino acid sequences [18] to the genome sequence. We found that the genes coding for Mrd and Mcc were arranged linearly and organized in one transcription unit (Fig 1). The *mrd* gene (1581 bp) was separated by 56 nucleotides from *mcc* (696 bp). The genes were flanked by a transposase gene 3297 nucleotides upstream of *mrd* separated from *mrd* by two pseudogenes and GTP cyclohydrolase gene 505 nucleotides downstream of *mcc*. Both flanking genes have the same orientation as *mrd* and *mcc*.

Putative promoter sites were found in close proximity to the predicted start codon. The sequences found 75 to 97 bp upstream of the translation start codon are similar to the consensus promoter sequences typically found -10 to -35 from the transcription start site. Furthermore, two transcription factor binding sites are predicted in this region, supporting the hypothesis that the promoter is a common regulatory element of the redox operon. A potential ρ-independent transcriptional terminator (energy of terminator -8.9) was found 75 nucleotides downstream of *mcc*. A second potential transcriptional terminator (terminator energy -9.4) is located in the spacer between the two genes and partially overlapped the *mcc* gene. The extra transcription termination signal located between the genes in the operon implies a complex regulation of the redox system at the transcriptional level.

### Evolution of the methacrylate redox system

**Fig 2:**
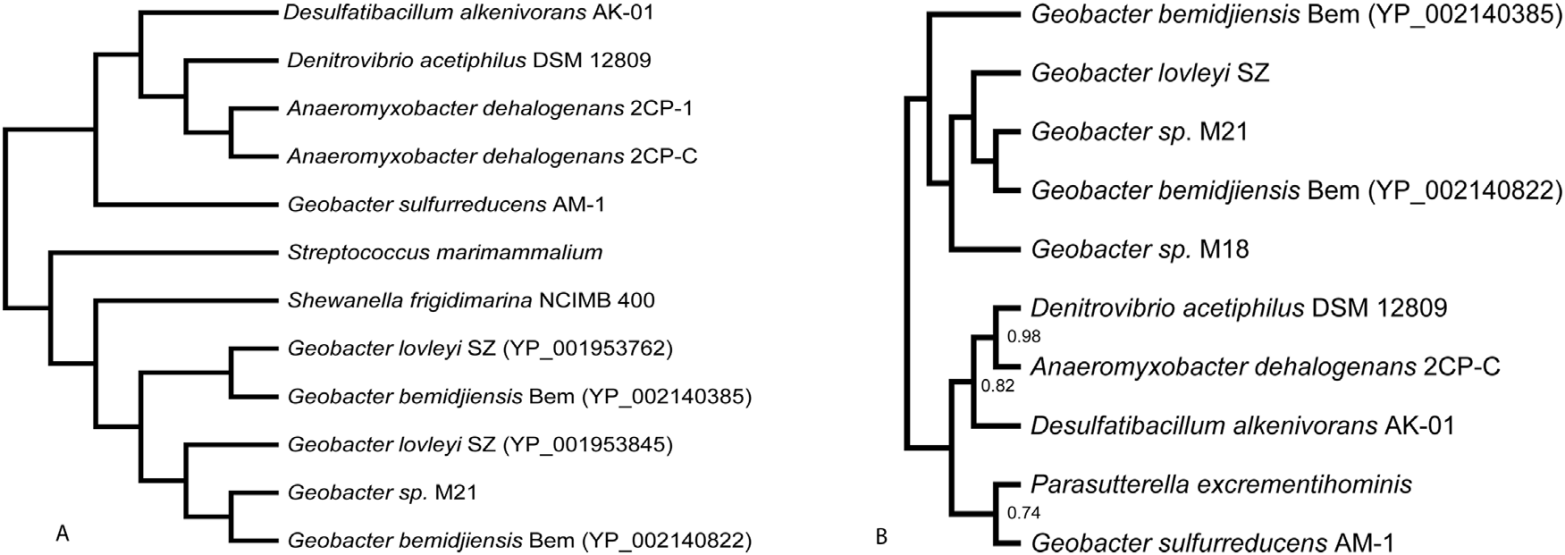
Phylogeny reconstructions for Mrd (A) and Mcc (B) homologs. Unrooted trees are shown with posterior probabilities. Unlabeled nodes have a posterior probability of 1. The following sequences were used. A (Mrd): *D*. *alkenivorans* AK-01 (YP_002429921.1), *D*. *acetiphilus* DSM 12809 (YP_003505239.1), *A*. *dehalogenans* 2CP-1 (YP_002492269.1), *A*. *dehalogenans* 2CP-C (YP_465303.1), *S*. *marimammalium* (WP_018370472.1), *S*. *frigidimarina* NCIMB 400 (YP_751265.1), *G*. *lovleyi* SZ (YP_001953845.1, YP_001953762.1), *G*. *bemidjiensis* Bem (YP_002140822.1, YP_002140385.1), *Geobacter* sp. M21 (YP_003023900.1). B (Mcc): *P*. *excrementihominis* YIT 11859 (WP_008864032.1), *D*. *alkenivorans* AK-01 (YP_002429920.1), *D*. *acetiphilus* DSM 12809 (YP_003505238.1), *A*. *dehalogenans* 2CP-C (YP_465304.1), *G*. *bemidjiensis* Bem (YP_002140822.1, YP_002140385.1), *G*. *lovleyi* SZ (YP_001953845.1), *G*. sp. M18 (YP_004200524.1), *G*. sp. M21 (YP_003023900.1).

To elucidate the evolutionary history of the methacrylate redox system, we searched for orthologues of *mrd* and *mcc*. First, we searched for homologs in the eleven *Geobacter* genomes available in GenBank. For *mrd* the closest homologs by protein sequence divergence were found in three strains: *G*. *lovleyi* SZ (YP_001951186.1, YP_001953845.1, YP_001953762.1), *G*. *bemidjiensis* Bem (YP_002140822.1, YP_002140385.1) and *Geobacter* sp. M21 (YP_003023900.1) (Table 1, Fig 2a). One of the homologs from *G*. *lovleyi* SZ, capable of chlororespiration, was the only protein from this list (YP_001951186.1) that does not contain the heme-binding sites CXXCH. Other homologous sequences found in *Geobacter* genomes have 4 heme-binding sites and different regions of their sequence are homologous to either Mrd or Mcc from the methacrylate redox system of *G*. *sulfurreducens* AM-1 (Tables 1 and 2; Fig 2a and 2b). Homology of Mcc from *G*. *sulfurreducens* AM-1 was observed for N-terminal amino acid sequence of *Geobacter* species flavocytochromes (usually 125 amino acids from the N-terminus). Sequence identity of Mrd with the flavocytochromes was higher (see column 5 of Tables 1 and 2) than for the region homologous to Mcc, and found in the C-terminal region (usually between the 140^th^ and 590^th^ amino acids). Thus, the methacrylate redox system homologs of bacteria of the genus *Geobacter* are often present as one multifunctional flavoprotein, combining functions of electron delivery and catalysis of reduction.

**Table 1:** Homologs of methacrylate reductase (Mrd).

Class	Species	GenBank accession number	Annotated function	% Similarity / Identity	Length of alignment with Mrd, e-value	Length, calculated Mr (kDa) of immature protein	Type of cleavable signal peptide (length)	Tat-motif	Heme-binding sites CXXCH
**Δ-Proteobacteria**	***Geobacter sulfurreducens* AM-1**		**methacrylate reductase**	**100/100**		**526 aa 57.2 kDa**	**Tat (55 aa)**	**RRDFLK**	**no**
**Δ-Proteobacteria**	***Anaeromyxobacter* sp. K**	**YP_002134140.1**	**flavocytochrome *c***	**78/64**	**96, 0.0**	**515 aa 55.5 kDa**	**Tat (38 aa)**	**RRAMLK**	**no**
**Δ-Proteobacteria**	***Anaeromyxobacter dehalogenans* 2CP-1**	**YP_002492269.1**	**flavocytochrome *c***	**78/64**	**96, 0.0**	**515 aa 55.6 kDa**	**Tat (38 aa)**	**RRAMLK**	**no**
**Δ-Proteobacteria**	***Anaeromyxobacter dehalogenans* 2CP-C**	**YP_465303.1**	**flavocytochrome *c***	**78/62**	**96, 0.0**	**515 aa 55.7 kDa**	**Tat (38 aa)**	**RRAILK**	**no**
**Δ-Proteobacteria**	***Desulfatibacillum alkenivorans* AK-01**	**YP_002429921.1**	**flavocytochrome *c***	**78/65**	**95, 0.0**	**511 aa 54.9 kDa**	**Tat (42 aa)**	**RRSVIK**	**no**
**Deferribacteres**	***Denitrovibrio acetiphilus* DSM 12809**	**YP_003505239.1**	**flavocytochrome *c***	**78/62**	**95, 0.0**	**507 aa 55.0 kDa**	**Tat (40 aa)**	**RRGLLQ**	**no**
**Δ-Proteobacteria**	***Geobacter lovleyi* SZ**	**YP_001951186.1**	**flavocytochrome *c***	**53/39**	**96, 8e** ^**-97**^	**517 aa 56.0 kDa**	**Tat (43 aa)**	**RRSFLK**	**no**
**Δ-Proteobacteria**	***Geobacter lovleyi* SZ**	**YP_001953845.1**	**flavocytochrome *c***	**54/39**	**87, 2e** ^**-71**^	**596 aa 63.3 kDa**	**Sec (25 aa)**	**no**	**4**
**Δ-Proteobacteria**	***Geobacter lovleyi* SZ**	**YP_001953762.1**	**flavocytochrome *c***	**51/38**	**90, 5e** ^**-72**^	**589 aa 61.7 kDa**	**Sec (26 aa)**	**no**	**4**
**Δ-Proteobacteria**	***Geobacter bemidjiensis* Bem**	**YP_002140822.1**	**flavocytochrome *c***	**55/40**	**88, 1e** ^**-78**^	**598 aa 63.3 kDa**	**Sec (25 aa)**	**no**	**4**
**Δ-Proteobacteria**	***Geobacter bemidjiensis* Bem**	**YP_002140385.1**	**flavocytochrome *c***	**51/38**	**88, 5e** ^**-72**^	**591 aa 61.5 kDa**	**Sec (21–27 aa)**	**no**	**4**
**Δ-Proteobacteria**	***Geobacter* sp. M21**	**YP_003023900.1**	**flavocytochrome *c***	**55/40**	**88, 1e** ^**-78**^	**598 aa 63.2 kDa**	**Sec (25 aa)**	**no**	**4**
**Γ-Proteobacteria**	***Shewanella frigidimarina* NCIMB 400**	**YP_749210.1**	**flavocytochrome *c***	**55/38**	**95, 8e** ^**-91**^	**510 aa 54.9 kDa**	**Tat (35 aa)**	**RRHFLK**	**no**
**Γ-Proteobacteria**	***Shewanella frigidimarina* NCIMB 400**	**YP_751265.1 (Ifc**_**3**_ **)**	**flavocytochrome *c***	**53/36**	**86, 3e** ^**-76**^	**588 aa 63 kDa**	**Sec (22 aa)**	**no**	**4**
**Γ-Proteobacteria**	***Shewanella frigidimarina* NCIMB 400**	**YP_751192.1**	**flavocytochrome *c***	**50/34**	**93, 1e** ^**-64**^	**507 aa 53.8 kDa**	**Tat (34 aa)**	**RRNIIK**	**no**
**Γ-Proteobacteria**	***Shewanella oneidensis* MR-1**	**NP_716599.1 (Fcc**_**3**_ **)**	**periplasmic fumarate reductase FccA**	**54/38**	**86, 2e** ^**-72**^	**596 aa 62.4 kDa**	**Sec (25 aa)**	**no**	**4**
**Γ-Proteobacteria**	***Shewanella oneidensis* MR-1**	NP_720136.1*	**urocanate reductase SO_4620**	**39/36**	**89, 2e** ^**-83**^	**582 aa 62.2 kDa**	**Sec (30 aa)**	**no**	**no**
**Γ-Proteobacteria**	***Shewanella frigidimarina* NCIMB 400**	Q07WU7.2*	**Periplasmic fumarate reductase; flavocytochrome c**	**51/35**	**84, 2e** ^**-63**^	**596 aa 63.0 kDa**	**Sec (25 aa)**	**no**	**4**
**Е-Proteobacteria**	***Wolinella succinogenes* DSM 1740**	NP_906388.1*	**flavocytochrome *c* flavin subunit FccA**	**51/35**	**87, 2e** ^**-67**^	**515 aa 55.9 kDa**	**Tat (34 aa)**	**RRDLIK**	**no**

* The last three proteins in the table have lower sequence similarity with methacrylate reductase. They were included in the table as they have been characterized biochemically.

**Table 2:** Homologs of cytochrome *c* (Mcc).

Class	Species	GenBank accession number	Annotated function	% Similarity / Identity	Length of alignment with Mcc, e-value	Length, calculated Mr (kDa) of immature protein	Type of cleavable signal peptide (length)	Heme-binding sites CXXCH
**Δ-Proteobacteria**	***Geobacter sulfurreducens* AM-1**		**cytochrome *c***	**100/100**		**231 aa**	**Sec (23 aa)**	**4–7**
**Δ-Proteobacteria**	***Anaeromyxobacter* sp. K**	**YP_002134139.1**	**hypothetical protein AnaeK_1781**	**55/42**	**84, 9e** ^**-40**^	**221 aa 22.9 kDa**	**Sec (21–24 aa)**	**7**
**Δ-Proteobacteria**	***Anaeromyxobacter dehalogenans* 2CP-1**	**YP_002492268.1**	**hypothetical protein A2cp1_1860**	**56/42**	**85, 2e** ^**-39**^	**221 aa 22.9 kDa**	**Sec (24 aa)**	**7**
**Δ-Proteobacteria**	***Anaeromyxobacter dehalogenans* 2CP-C**	YP_465304.1*	**hypothetical protein Adeh_2097**	**56/42**	**84, 3e** ^**-47**^	**233 aa 24.1 kDa**	**Sec (24 aa)**	**7**
**Δ-Proteobacteria**	***Desulfatibacillum alkenivorans* AK-01**	**YP_002429920.1**	**hypothetical protein Dalk_0747**	**51/40**	**44, 2e** ^**-15**^	**109 aa 12.1 kDa**	**no**	**4**
**Deferribacteres**	***Denitrovibrio acetiphilus* DSM 12809**	YP_003505238.1*	**hypothetical protein Dacet_2522**	**48/36**	**94, 1e** ^**-32**^	**208 aa 22.9 kDa**	**Sec (18 aa)**	**7**
**Δ-Proteobacteria**	***Geobacter lovleyi* SZ**	**YP_001953845.1**	**flavocytochrome *c***	**54/44**	**42, 1e** ^**-13**^	**596 aa 63.3 kDa**	**Sec (25 aa)**	**4**
**Δ-Proteobacteria**	***Geobacter lovleyi* SZ**	**YP_001953762.1**	**flavocytochrome *c***	**50/39**	**42, 4e** ^**-10**^	**589 aa 61.7 kDa**	**Sec (26 aa)**	**4**
**Δ-Proteobacteria**	***Geobacter bemidjiensis* Bem**	**YP_002140822.1**	**flavocytochrome *c***	**48/42**	**41, 2e** ^**-11**^	**598 aa 63.3 kDa**	**Sec (25 aa)**	**4**
**Δ-Proteobacteria**	***Geobacter bemidjiensis* Bem**	**YP_002140385.1**	**flavocytochrome *c***	**59/44**	**35, 1e** ^**-12**^	**591 aa 61.5 kDa**	**Sec (21–27 aa)**	**4**
**Δ-Proteobacteria**	***Geobacter* sp. M18**	**YP_004200524.1**	**flavocytochrome *c***	**62/51**	**35, 3e** ^**-14**^	**584 aa 60.7 kDa**	**Sec (22–23 aa)**	**4**
**Δ-Proteobacteria**	***Geobacter* sp. M21**	**YP_003023900.1**	**flavocytochrome *c***	**48/42**	**41, 7e** ^**-11**^	**598 aa 63.2 kDa**	**Sec (25 aa)**	**4**
**Γ-Proteobacteria**	***Shewanella frigidimarina* NCIMB 400**	**Q07WU7.2 (Fcc**_**3**_ **)**	**fumarate reductase flavoprotein subunit; flavocytochrome *c***	**53/38**	**48, 6e** ^**-11**^	**596 aa 63.0 kDa**	**Sec (25 aa)**	**4**
	***Shewanella frigidimarina* NCIMB 400**	**YP_751265.1 (Ifc**_**3**_ **)**	**flavocytochrome c**	**51/39**	**40,1e** ^**-09**^	**588 aa 63 kDa**	**Sec (22 aa)**	**4**
**Γ-Proteobacteria**	***Shewanella frigidimarina* NCIMB 400**	**YP_751191.1**	**tetraheme cytochrome *c***	**50/34**	**41, 6e** ^**-07**^	**122 aa 13.9 kDa**	**Sec (22 aa)**	**4**
**Γ-Proteobacteria**	***Shewanella oneidensis* MR-1**	**NP_716599.1 (Fcc**_**3**_ **)**	**periplasmic fumarate reductase FccA**	**59/46**	**35, 3e** ^**-10**^	**596 aa 62.4 kDa**	**Sec (25 амк)**	**4**
**Β-Proteobacteria**	***Parasutterella excrementihominis* YIT 11859**	**WP_008864032.1**	**hypothetical protein HMPREF9439_01147**	**50/33**	**80, 7e** ^**-25**^	**208 aa 22.7 kDa**	**Sec (19 aa)**	**6–7**
**Е-Proteobacteria**	***Wolinella succinogenes* DSM 1740**	NP_906387.1**	**flavocytochrome *c* heme subunit**	**44/28**	**51, 0.002**	**146 aa 16.6 kDa**	**Sec (26 aa)**	**4**

*Annotated by us.

**The last protein in the table has lower sequence similarity than others. It was included because it has been characterized biochemically.

A diversity of other cytochrome *c* protein sequences were found to be coded in *Geobacter* genomes [30–36], which were much more diverged than the *Geobacter* homologs we considered in our phylogenetic analysis. None of these distantly related genes were considered in our analysis.

Homologs of both Mrd and Mcc with higher sequence identity were found outside the *Geobacter* genus in a few species with a broad phylogenetic distribution, indicating a complex evolutionary origin of these proteins in *G*. *sulfurreducens* AM-1. The distribution of Mrd homologs varied across bacterial clades. The closest of the identifiable Mrd homologs (78% similarity; Table 1, Fig 2a), which were annotated as flavoproteins, were from *δ-Proteobacteria*: *Anaeromyxobacter dehalogenans* 2CP-C (YP_465303.1), *A*. *dehalogenans* 2CP-1 (YP_002492269.1), *A*. *sp*. K (YP_002134140.1) and *Desulfatibacillum alkenivorans* AK-01 (YP_002429921.1) and *Deferribacteres*: *Denitrovibrio acetiphilus* DSM 12809 (YP_003505239.1).

Interestingly, the same species that harbor the closest homologs of Mrd also have the closest homologs of Mcc (48–58% similarity; Table 2, Fig 2b): *A*. *dehalogenans* 2CP-C (YP_465304.1), *A*. *dehalogenans* 2CP-1 (YP_002492268.1), *A*. *sp*. K (YP_002134139.1), *D*. *alkenivorans* AK-01 (YP_002429920.1), *D*. *acetiphilus* DSM 12809 (YP_003505238.1). We annotated them as multiheme cytochrome *c* (Table 2). The redox systems of these organisms were represented by two proteins with their genes organized in one transcriptional unit. The only exception to having a close homologue of both Mdr and Mcc was *Parasutterella excrementihominis* YIT 11859 (β-Proteobacterium) that had a close homolog only of Mcc (WP_008864032.1, Table 2, Fig 2b).

To confirm that the *G*. *sulfurreducens* AM-1 Mrd and Mcc homologs found in other *Geobacter* species are not their direct orthologues, we performed a phylogenetic analysis of the homologs, including several of the sequences from the *Geobacter* genus that were most similar to Mrd of *G*. *sulfurreducens* AM-1. The analysis showed that *G*. *sulfurreducens* AM-1 Mrd and Mcc share a closer common ancestor with sequences from distant clades of bacteria, confirming that the methacrylate redox system genes, *mrd* and *mcc*, were likely acquired by *G*. *sulfurreducens* AM-1 through recent horizontal gene transfer and that their orthologues are not present in the sequenced *Geobacter* genomes (Fig 2a and 2b).

### Products of the methacrylate redox system genes

**Fig 3:**
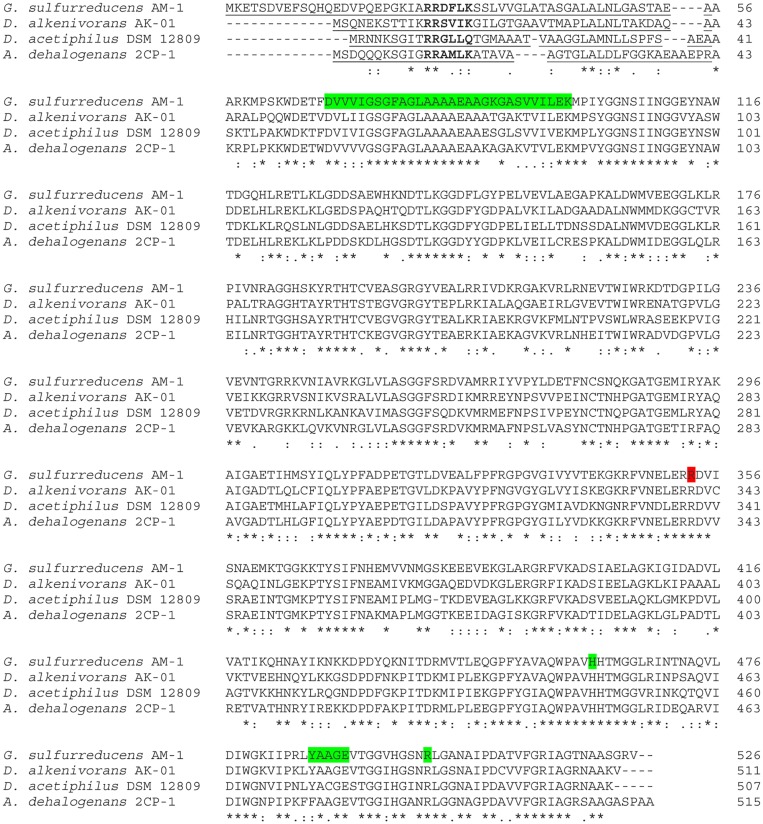
Multiple protein sequence alignment of Mrd coded in *Geobacter sulfurreducens* AM-1 and its closest flavocytochrome *c* homologs from *Desulfatibacillum alkenivorans* AK-01 (YP_002429921.1); *Denitrovibrio acetiphilus* DSM 12809 (YP_003505239.1) and *Anaeromyxobacter dehalogenans* 2CP-1 (YP_002492269.1). Amino acid sequences of Mrd homologs (YP_002134140.1, YP_002492269.1, YP_465303.1) of all three mentioned representatives of the genus *Anaeromyxobacter* are very similar. Therefore, we used the sequences of the Mrd homolog only from *A*. *dehalogenans* 2CP-1 (YP_002492269.1) as one representative of the genus. Cleavable signal peptides of Tat type are underlined; the Tat motif is shown in bold. Conserved pyrophosphate-binding sites and amino acids presumably involved in catalysis are highlighted in green. Probable proton donor is marked in red.

The protein coded by *mrd* has 526 amino acids (Mr 57.2 kDa). The N-terminal amino acid sequence contains a 55 amino acid-long signal peptide with the Tat-motif RRDFLK in position 25 (Fig 3, Table 1). Thus, the mature protein is predicted to contain 471 amino acids (estimated Mr 51.4 kDa). Previous results have shown that the mature Mrd has 1 mol FAD [18]; therefore, the Mr of the mature Mrd with FAD should be 52.2 kDa, which is consistent with experimental data. We validated the start and flanking regions of *mrd* by Sanger sequencing of both strands, which were identical to the sequences obtained through the next generation sequencing of the entire genome. Thus, the unusually long predicted signal peptide was confirmed not to result from sequencing or assembly error.

**Fig 4:**
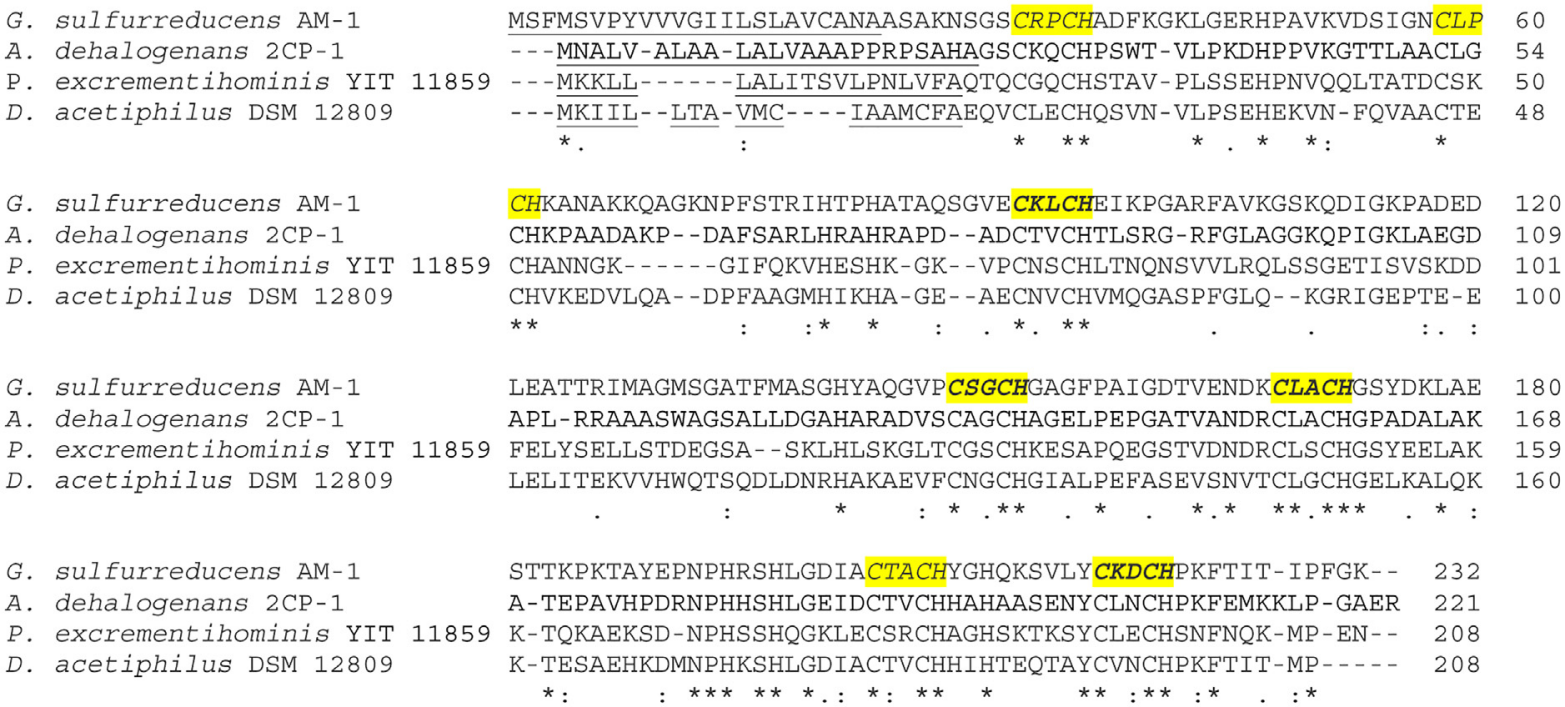
Multiple protein sequence alignment of Mcc from *Geobacter sulfurreducens* AM-1 and its cytochrome *c* homologs from *Anaeromyxobacter dehalogenans* 2CP-1 (YP_002492268.1); *Parasutterella excrementihominis* YIT 11859 (WP_008864032.1) and *Denitrovibrio acetiphilus* DSM 12809 (YP_003505238.1). Amino acid sequences of Mcc homologs (YP_002134139.1, YP_002492268.1, YP_465304.1) of all three representatives of the genus *Anaeromyxobacter* are very similar. We used the sequences of the Mcc homolog only from *A*. *dehalogenans* 2CP-1 (YP_002492268.1) as one representative of the genus. The amino acid sequences of protein YP_002429920 from *D*. *alkenivorans* AK-01 (YP_002429920.1), which is not shown here, is much shorter than those included, which makes it difficult to analyze. Cleavable signal peptides of Sec type are underlined. Heme-binding sites are marked in italics and highlighted in yellow. Sites, detected by GENERUNR program, are in bold.

The *mcc* gene codes for a protein 231 amino acids long (Mr 24.5 kDa). The N-terminal region contains a shorter Sec-type signal peptide of 23 amino acids (Fig 4, Table 2) with the mature protein predicted to have 208 amino acids (Mr 22.1 kDa). Previous experiments showed that the mature Mcc had 4 mol of heme *c* and a Mr of nearly 30 kDa [16]. Consistent with these results, we found four heme-binding motifs CXXCH [37] with the GENE RUNNER program. The Mr of a mature Mcc with 4 hemes is 24.8 kDa, substantially lower than expected. A visual analysis of the Mcc sequence revealed three more heme-binding motifs CXXCH, which brought the Mr of the mature Mcc with 7 hemes to 27.9 kDa (Fig 4).

The closest of the identifiable homologs of Mrd (Fig 2a) are likely FAD-binding proteins and flavocytochromes *c*, as indicated by the conserved phosphate-binding regions of N-termini (Fig 3). The phosphate-binding site is typical for all FAD- and NAD(P)H-dependent oxidoreductases: xhxhGxGxxGxxxhxxh(x)_8_hxhE(D), where x—any amino acid, h—hydrophobic amino acid [38]. In the case of Mrd this site was located between amino acids 69 and 98 of the immature protein (Fig 3). The central part of the consensus, GxGxxG, is a glycine-rich part of the loop, linking the first β-sheet in the Rossmann fold with the first α-helix directed to the pyrophosphate residue for charge compensation. Generally this motif has β-strand-turn-β-strand structure and forms a flexible clamp, surrounding and anchoring the pyrophosphate of FAD [39]. Another conservative FAD-binding site, which is an eleven amino acid segment T(S)xxxxxF(Y)hhGD(E) [40], was present in amino acid sequences of Mrd and its homologs. The site was slightly truncated, without the first threonine while all other amino acids were present (487–491).

The heme-binding sites of Mcc homologs identified by the phylogenetic analysis (Fig 2b) are shown in Fig 4. Their presence suggests that these homologs, not annotated as having any function, are cytochromes *c*, containing four heme-binding sites (YP_002429920.1) in *D*. *alkenivorans* AK-01 or seven heme-binding sites in the other species.

The Mrd sequence of *G*. *sulfurreducens* AM-1 had a higher level of similarity with its homologs (Table 1) than Mcc of *G*. *sulfurreducens* AM-1 (Table 2). This observation is consistent with a relatively poor conservation of cytochromes *c* [36] and probably with evolutionary early origin of the flavin-containing Mrd homologs.

## Discussion

**Fig 5:**
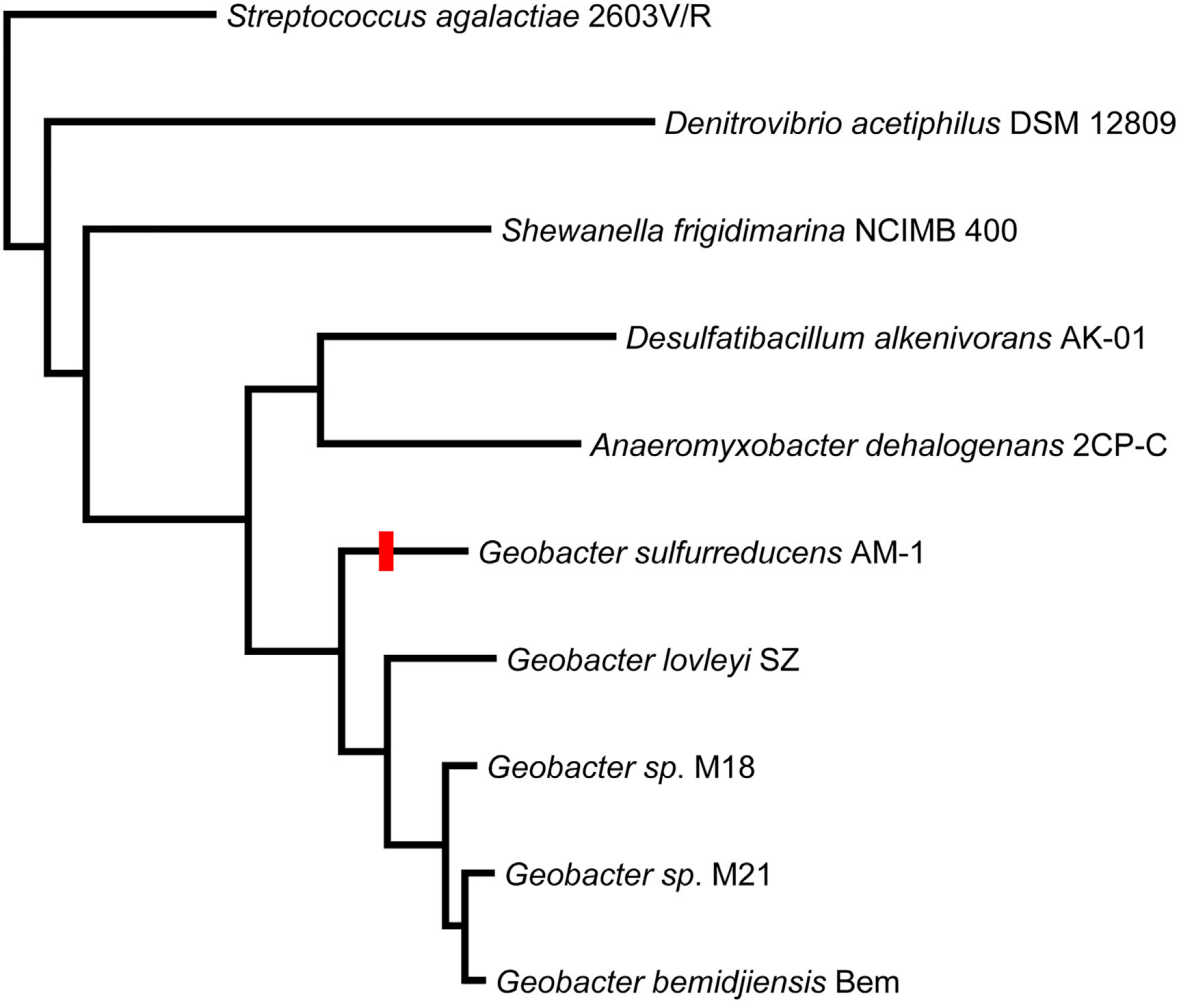
An unrooted phylogeny reconstruction of 16s RNA from the strains coding for *mrd* and *mcc* homologs or from their closest relatives. The branch on which the horizontal gene transfer of the operon carrying the *mrd* and *mcc* genes has occurred is indicated by a red mark.

The methacrylate redox system genes in the genome of *G*. *sulfurreducens* AM-1 appear to be arranged in a single operon. The clear absence of orthologs in the genomes of several other *Geobacter* genomes, coupled with a lack of closely-related orthologs in genomes of bacteria from any other closely related genus, strongly suggests that the methacrylate redox system genes were acquired recently by the *G*. *sulfurreducens* AM-1 strain (Fig 5). The intriguing similarity of the phylogenetic distribution of the closely related homologs of both genes, *mrd* and *mcc*, suggests that these two genes tend to be horizontally transferred together, confirming their close functional relationship. The high congruence of the evolutionary history of the *mrd* and *mcc* genes is consistent with their organization into a single operon and confirms their joint functional role.

Unfortunately, for most of the identified homologs experimental data of their enzyme specificity are not available. Such lack of experimental data precludes us from understanding whether or not the acquisition of the methacrylate reducing function occurred before or after the horizontal gene transfer. Furthermore, even the closest of the identified homologs were evidently too diverged to be identified as the origin of the horizontal gene transfer. This conclusion is based on the observation of the divergence of Mrd and Mcc sequences from their closest homologs in comparison to the high similarity of genomes of different *Geobacter* species.

Nevertheless, some experimentally characterized proteins can be distinguished among the homologs of the methacrylate redox system. The characterized homologs of Mrd include flavoprotein FccA (NP_906388.1) from *Wolinella succinogenes* [41], an urocanate reductase SO_4620 (NP_720136.1; [42]) and periplasmic fumarate reductases Fcc_3_ (Q07WU7.2; [9, 10,43]), Ifc_3_ (YP_751265.1; [11,12]) and Fcc_3_ (NP_716599.1; [13–15]) from bacteria of the genus *Shewanella* (Table 1). *Shewanella*’s periplasmic fumarate reductases are cytochromes *c* homologs as well (Table 2). Therefore, the methacrylate redox system and its homologs reduce the double bonds of unsaturated organic compounds (such as acrylate, methacrylate, urocanate, fumarate), using them as terminal acceptors of reducing equivalents. None of the species or strains described previously are known to grow by respiration of methacrylate.

Conserved amino acids (histidine-461 and arginines—R501 and R353, Fig 3), found in the Mrd sequence, may stabilize the transition state during catalysis by providing delocalization of the negative charge of the intermediate carbanion, in a similar manner as in *Shewanella* fumarate reductases [16]. Point mutagenesis showed that the arginine homologous to R353 of Mrd is the proton donor for the carbanion [44]. The fumarate reductase arginine homologous to R501 in Mrd interacts through its guanidino group with both oxygen atoms of a carboxyl group of succinate, positioning it parallel to the isoalloxazine ring [16]. Mrd does not have two other conserved residues that interact with succinate or fumarate. It has a tryptophan instead of histidine at position 311 and valine instead of serine or threonine at position 324. Since these amino acids are also involved in substrate binding, it is possible that their absence is due to substrate specificity of Mrd of *G*. *sulfurreducens* AM-1.

Biogenesis of chromoproteids of the methacrylate redox system probably occurs via different mechanisms. The immature Mrd protein has a longer and less hydrophobic Tat-type signal peptide sequence (Table 1, Fig 3), characteristic for *Bacteria*, *Archaea* and chloroplast proteins. Such proteins are transported through the membrane after folding [45]. A Sec-type signal peptide sequence was found in the immature Mcc protein (Table 2, Fig 4). Such proteins are translocated across the membrane before the acquisition of tertiary structure [45,46], with heme attachment occurring in the periplasm [37,46]. Thus, both the Tat- and Sec-type secretory mechanisms are likely to be required for maturation of the methacrylate redox system proteins.

Genes of the methacrylate redox system components of *G*. *sulfurreducens* AM-1 are organized similarly to genes for their closest homologs in four representatives of *δ-Proteobacteria* and one representative of *Deferribacteres* (see RESULTS). Thus, it is possible that these organisms may also either be able to grow using methacrylate as a terminal electron acceptor or at least show some methacrylate-reducing activity.

The methacrylate redox system is representative of a comprehensive family of flavocytochromes *c* and flavoproteins with reducing properties. These reducing complexes probably use a natural substrate, for example, acrylate produced by marine bacteria [26,47]. The rates of reduction of acrylate and methacrylate by the methacrylate redox system are comparable [18] supporting the hypothesis of the use of some natural substrate by these proteins. Methacrylate reduction may be an additional characteristic of this redox system.

Components of the methacrylate redox system from *G*. *sulfurreducens* AM-1 and lyase of dimethylsulphoniopropionate (DMSP) of DddY-type from marine microorganisms have some similar features: 1) a distribution in certain groups of proteobacteia, 2) gene organization with cytochrome *c* genes adjacent to the enzyme genes (reductase or lyase) and 3) presence of cleavable signal peptide in the immature enzymes. The enzyme DddY catalyzes the cleavage of DMSP to the volatile compound dimethyl sulphide (DMS) and the toxic acrylate [47]. We suggest that the reductase evolved to transform the toxic acrylate, formed by lyases, to a less toxic compound. These cytochromes *c*, whose genes are located near the reductase or lyase genes, may be homologous.

The methacrylate redox system evolved from a cytochrome *c* and a flavoprotein. These proteins were recently acquired by horizontal gene transfer by *G*. *sulfurreducens* AM-1 either before or after the evolution of the substrate specificity. Furthermore, these proteins likely constitute an adaptive mechanism to allow growth in sludge microbial communities, in particular, in wastewater of plastic manufacture factories.

## Experimental Procedures

The *object of investigation* was anaerobic bacterium *G*. *sulfurreducens* AM-1 from the culture collection of Laboratory of microorganisms adaptation at the Institute of Biochemistry and Physiology of Microorganisms (Pushchino, Russia).

The *subject of investigation* was the operon containing genes *mrd* and *mcc* of the methacrylate redox system of *G*. *sulfurreducens* AM-1.

### Genome sequencing

The draft genome sequences were obtained by pair-end library and mate pair library sequences by Illumina HiSeq 2000. The resulting contigs were submitted to GenBank under the accession numbers of CP010430.

### Genome assembly

The genome was assembled de novo using SOAPdenovo [48], Velvet [49] and SPAdes Genome Assembler [50]. The quality of assembly was estimated by running QUAST [51] and by aligning of the contigs to the full genomes of *Geobacter sulfurreducens* available in GenBank: *Geobacter sulfurreducens* KN400 and *Geobacter sulfurreducens* PCA. The alignments were done with Mauve [52].

The contigs obtained by SPAdes turned out to be the best. Nevertheless, SPAdes failed to assemble the genome into one sequence. We used SSPACE [53] for scaffolding. This allowed us to obtain the genome as just one contig. After this we applied GapFiller [54] for closing gaps.

### Sequence analysis

*Detection of the mrd and mcc* genes of the bacterium *G*. *sulfurreducens* AM-1 and *comparative amino acid analysis* were performed with the BLAST program [55] from the National Center of Biotechnology Information server, National Library of Medicine, USA (NCBI; http://www.ncbi.nlm.nih.gov).

*Analysis of nucleotide sequences* of the studied operon was carried out using the Vector NTI program [56]. The presence and types of *promoters and terminators* were detected with a series of programs, available on the site http://linux1.softberry.com.

*The sequencing of the mrd start and mrd flanking regions* was performed by the Sanger method with oligonucleotide primers FA2 (5’-ACGCTTCTCAACCAGACCGG) and RA2 (5’-CATCGGTCCAAGCGTTATATTCAC). Amplification for the nucleotide sequencing was performed by the PCR method using oligonucleotide primers—FG1 (5’-CAGAACAGGCCACGCTTTGC) and RG1 (5’- GTGCGGTACTTGCTGTGCCC).

All amino acid sequences of proteins and nucleotide sequences of genes are available in the *Databases* GenBank, Gene, Genome, Nucleotide, Protein from the server of the NCBI.

*Determination of the cleavable signal peptides* was conducted with the programs PRED-TAT [45] and SignalP [57], available on servers of the Department of Computer Science and Biomedical Informatics, University of Central Greece, Lamia, Greece (http://www.compgen.org) and the Center for Biological Sequence Analysis, Department of Systems Biology, Technical University of Denmark, Lyngby, Denmark (http://www.cbs.dtu.dk).

Program GENERUNR (http://www.generunner.net) was used for the *detection of conserved amino acid sequences and calculation of molecular weight*. The number of hemes in homological proteins was predicted as number of heme-binding sites CXXCH (where C is cysteine, H is histidine, X is any amino acid) [37].

*Multiple protein sequence alignment* of methacrylate redox system components and their homologs was performed with MUSCLE [58]. Phylogenies were reconstructed using the MrBayes v3.2 program [59], with mcmc = 3000000 and burnin = 2500 for sump and sumt.

## References

[1] Arkhipova OV, Akimenko VK. Unsaturated organic acids as terminal electron acceptors for reductase chains of anaerobic bacteria. Microbiology 2005;74: 629–239. 16400981

[2] Ackrell BAC, Johnson MK, Gunsalus RP, Cecchini G. Structure and function of succinate dehydrogenase and fumarate reductase Chemistry and biochemistry of flavoenzymes Ed. Muller F. Boca Raton, Florida: CRC Press 1992;3: 229–297.

[3] Hägerhäll C. Succinate:quinone oxidoreductases. Variations on a conserved theme. Biochim Biophys Acta 1997;1320: 107–141. 9210286 10.1016/s0005-2728(97)00019-4

[4] Lemos RS, Fernandes AS, Pereira MM, Gomes CM, Miguel M. Quinol:fumarate oxidoreductases and succinate: quinone oxidoreductases: phylogenetic relationships, metal centres and membrane attachment. Biochim Biophys Acta 2002;1553: 158–170. 11803024 10.1016/s0005-2728(01)00239-0

[5] Iverson T. Catalytic mechanisms of complex II enzymes: A structural perspective. Biochim Biophys Acta 2013;1827: 648–657. 10.1016/j.bbabio.2012.09.008 22995215 PMC3537904

[6] Kröger A. Bacterial electron transport to fumarate Diversity of bacterial respiratory systems Ed. Knowles C.J. Boca Raton, Florida: CRC Press 1980;1–17.

[7] Kröger A, Geisler V, Lemma E, Theis F, Lenger R. Bacterial fumarate respiration. Arch Microbiol 1992;158: 311–314.

[8] Kröger A, Biel S, Simon J, Gross R, Unden G, Lancaster CR. Fumarate respiration of *Wolinella succinogenes*: enzymology, energetics and coupling mechanism. Biochim Biophys Acta 2002;1553: 23–38. 11803015 10.1016/s0005-2728(01)00234-1

[9] Pealing SL, Black AS, Manson FDC, Ward FB, Chapman SK, Reid GA. Sequence of the gene encoding flavocytochrome *c* from *Shewanella putrefaciens*: a tetraheme flavoenzyme that is a soluble fumarate reductase related to the membrane-bound enzymes from other bacteria. Biochemistry 1992;32: 12132–12140. 10.1021/bi00163a023 1333793

[10] Morris CJ, Black AS, Pealing SL, Manson FDC, Chapman SK, Reid GA, et al Purification and properties of a novel cytochrome: flavocytochrome *c* from *Shewanella putrefaciens* . Biochem J 1994;302: 587–593. 8093012 10.1042/bj3020587 PMC1137268

[11] Dobbin PS, Butt JN, Powell AK, Reid GA, Richardson DJ. Characterization of a flavocytochrome that is induced during the anaerobic respiration of Fe^3+^ by *Shewanella frigidimarina* NCIMB 400. Biochem J 1999;342: 439–448. 10455032 PMC1220482

[12] Bamford V, Dobbin PS, Richardson DJ, Hemmings AM. Open conformation of a flavocytochrome *c* _*3*_ fumarate reductase. Nat Struct Biol 1999;6: 1104–1107. 10581549 10.1038/70039

[13] Tsapin AI, Vandenberghe I, Nealson KH, Scott JH, Meyer TE, Cusanovich MA, et al Identification of a small tetraheme cytochrome *c* and a flavocytochrome *c* as two of the principal soluble cytochromes *c* in *Shewanella oneidensis* strain MR1. Appl Environ Microbiol 2001;67: 3236–3244. 11425747 10.1128/AEM.67.7.3236-3244.2001 PMC93006

[14] Maier TM, Myers JM, Myers CR. Identification of the gene encoding the sole physiological fumarate reductase in *Shewanella oneidensis* MR-1. J Basic Microbiol 2003;43: 312–327. 12872312 10.1002/jobm.200390034

[15] Myers CR, Myers JM. Isolation and characterization of a transposon mutant of *Shewanella putrefaciens* MR-1 deficient in fumarate reductase. Lett Appl Microbiol 1997;25: 162–168. 9351256 10.1046/j.1472-765x.1997.00196.x

[16] Leys D, Tsapin AS, Nealson KH, Meyer TE, Cusanovich MA, Van Beeumen JJ. Structure and mechanism of the flavocytochrome *c* fumarate reductase of *Shewanella putrefaciens* MR-1. Nat Struct Biol 1999;6: 1113–1117. 10581551 10.1038/70051

[17] Galushko AS, Obraztsova AYa, Shtarkman NB, Laurinavichyus KS, Akimenko VK. An anaerobic acetate-oxidizing bacterium transforming methacrylic acid. Doklady Biological Sciences 1994;335: 122–123.

[18] Mikoulinskaia (Arkhipova) O, Akimenko V, Galushko A, Thauer R, Hedderich R. Cytochrome *c*-dependent methacrylate reductase from *Geobacter sulfurreducens* AM-1. Eur J Biochem 1999;263: 346–352. 10406941 10.1046/j.1432-1327.1999.00489.x

[19] Caccavo FJR, Lonergan DJ, Lovley DR, Davis M, Stolz JF, McInerney MJ. *Geobacter sulfurreducens sp*. *nov*., a hydrogen- and acetate-oxidizing dissimilatory metal-reducing microorganism. Appl Environ Microbiol 1994;60: 3752–3759. 7527204 10.1128/aem.60.10.3752-3759.1994 PMC201883

[20] Nevin KP, Holmes DE, Woodard TL, Hinlein ES, Ostendorf DW, Lovley DR. *Geobacter bemidjiensis* sp. nov. and *Geobacter psychrophilus* sp. nov., two novel Fe(III)-reducing subsurface isolates. Int J Syst Evol Microbiol 2005;55: 1667–1674. 16014499 10.1099/ijs.0.63417-0

[21] Sung Y, Fletcher KE, Ritalahti KM, Apkarian RP, Ramos-Hernández N, Sanford RA, et al *Geobacter lovleyi* sp. nov. strain SZ, a novel metal-reducing and tetrachloroethene-dechlorinating bacterium. Appl Environ Microbiol 2006;72: 2775–2782. 16597982 10.1128/AEM.72.4.2775-2782.2006 PMC1448980

[22] Amos BK, Sung Y, Fletcher KE, Gentry TJ, Wu W-M, Criddle CS, et al Detection and quantification of *Geobacter lovleyi* strain SZ: implications for bioremediation at tetrachloroethene- and uranium-impacted sites. Appl Environ Microbiol 2007;73: 6898–6904. 17827319 10.1128/AEM.01218-07 PMC2074934

[23] Galushko AS, Schink B. Oxidation of acetate through reactions of the citric acid cycle by *Geobacter sulfurreducens* in pure culture and in syntrophic coculture. 2000;Arch Microbiol 174: 314–321. 11131021 10.1007/s002030000208

[24] Lovley DR. Powering microbes with electricity: direct electron transfer from electrodes to microbes. Environ Microbiol Rep 2011;3: 27–35. 10.1111/j.1758-2229.2010.00211.x 23761228

[25] Galushko AS, Mikulinskaya (Arkhipova) OV, Laurinavichyus KS, Obraztsova AYa, Akimenko VK. Periplasmic location of methacrylate reductase in cells of the strictly anaerobic bacterial strain AM-1. Microbiology (Engl. Transl. Mikrobiologiya (Russia)) 1996;65: 432–435.

[26] Van der Maarel MJEC, van Bergeijk S, van Werkhoven AF, Laverman AM, Meijer WG, Stam WT, et al Cleavage of dimethylsulfoniopropionate and reduction of acrylate by *Desulfovibrio acrylicus* sp.nov. Arch Microbiol 1996;166: 109–115.

[27] Arkhipova OV, Chuvochina MS, Trutko SM. Cytochromes *c* of the anaerobic methacrylate reducer *Geobacter sulfurreducens* AM-1. Microbiology 2009;8: 296–303. 10.1186/1475-2875-8-296 19580156

[28] Arkhipova OV, Mikulinskaya GV, Galushko AS. Comparative analysis of the N-terminal sequence of *Geobacter sulfurreducens* AM-1 methacrylate reductase. Microbiology 2012;81: 555–564. 23234071

[29] Stickler M, Rhein T. Polymethacrylates Ulmann’s Encyclopedia of Industrial Chemistry Wiley-VCH Verlag GmbH & Co. KGaA, Weinheim 2012;29: 342–353.

[30] Methė BA, Nelson KE, Eisen JA, Paulsen IT, Nelson W, Heidelberg JF, et al Genome of *Geobacter sulfurreducens*: metal reduction in subsurface environments. Science 2003;302: 1967–1969. 14671304 10.1126/science.1088727

[31] Aklujkar M, Young ND, Holmes D, Chavan M, Risso C, Kiss HE, et al The genome of *Geobacter bemidjiensis*, exemplar for the subsurface clade of *Geobacter* species that predominate in Fe(III)-reducing subsurface environments. BMC Genomics 2010;11:490 10.1186/1471-2164-11-490 20828392 PMC2996986

[32] Nagarajan H, Butler JE, Klimes A, Qiu Y, Zengler K, Ward J, et al De novo assembly of the complete genome of an enhanced electricity-producing variant of *Geobacter sulfurreducens* using only short reads. PLoS ONE 2010;5 10.1371/journal.pone.0010922 PMC2882325 20544019

[33] Aklujkar M, Krushkal J, DiBartolo G, Lapidus A, Land ML, Lovley DR. The genome sequence of *Geobacter metallireducens*: features of metabolism, physiology and regulation common and dissimilar to *Geobacter sulfurreducens* . BMC Microbiology 2009;9:109 10.1186/1471-2180-9-109 19473543 PMC2700814

[34] Butler JE, Nelson DY, Aklujkar M, Lovley DR. Comparative genomic analysis of *Geobacter sulfurreducens* KN400, a strain with enhanced capacity for extracellular electron transfer and electricity production. BMC Genomics 2012;13:471 10.1186/1471-2164-13-471 22967216 PMC3495685

[35] Butler JE, Young ND, Lovley DR. Evolution from a respiratory ancestor to fill syntrophic and fermentative niches: comparative genomics of six *Geobacteraceae* species. BMC Genomics 2009;10:103 10.1186/1471-2164-10-103 19284579 PMC2669807

[36] Butler JE, Young ND, Lovley DR. Evolution of electron transfer out of the cell: comparative genomics of six *Geobacter* genomes. BMC Genomics 2010 11:40 10.1186/1471-2164-11-40 20078895 PMC2825233

[37] Li T, Bonkovsky HL, Guo J. Structural analysis of heme proteins: implications for design and prediction. BMC Structural Biology 2011;11 10.1186/1472-6807-11-13 PMC3059290 21371326

[38] Eschenbrenner M, Chlumsky LJ, Khanna P, Strasser F, Jorns MS. Organization of the multiple coenzymes and subunits and role of the covalent flavin link in the complex heterotetrameric sarcosine oxidase. Biochemistry 2001;40: 5352–5367. 11330998 10.1021/bi010101p

[39] Hanks SK, Hunter T. Protein kinases 6. The eukaryotic protein kinase superfamily: kinase (catalytic) domain structure and classification. FASEB J 1995;9: 576–596. 7768349

[40] Eggink G, Engel H, Vriend G, Terpstra P, Witholt B. Rubredoxin reductase of *Pseudomonas oleovorans*. Structural relationship to other flavoprotein oxidoreductases based on one NAD and two FAD fingerprints. J Mol Biol 1990;212: 135–142. 2319593 10.1016/0022-2836(90)90310-I

[41] Simon J, Gross R, Klimmek O, Ringel M, Kröger A. A periplasmic flavoprotein in *Wolinella succinogenes* that resembles the fumarate reductase of *Shewanella putrefaciens* . Arch Microbiol 1998;169: 424–433. 9560424 10.1007/s002030050593

[42] Bogachev AV, Bertsova YV, Bloch DA, Verkhovsky MI. Urocanate reductase: identification of a novel anaerobic pathway in *Shewanella oneidensis* MR-1. Mol Microbiol 2012;86:1452–1463. 10.1111/mmi.12067 23078170

[43] Gordon EHJ, Pealing SL, Chapman SK, Ward FB, Reid GA. Physiological function and regulation of flavocytochrome *c* _3_, the soluble fumarate reductase from *Shewanella putrefaciens* NCIMB 400. Microbiology 1998;144: 937–945. 9579067 10.1099/00221287-144-4-937

[44] Doherty MK, Pealing SL, Miles CS, Moysey R, Taylor P, Walkinshaw MD, et al Identification of the Active Site Acid/Base Catalyst in a Bacterial Fumarate Reductase: A Kinetic and Crystallographic Study. Biochemistry 2000;39: 10695–10701. 10978153 10.1021/bi000871l

[45] Bagos PG, Nikolaou EP, Liakopoulos TD, Tsirigos KD. Combined prediction of Tat and Sec signal peptides with Hidden Markov Models. Bioinformatics 2010;26: 2811–2817. 10.1093/bioinformatics/btq530 20847219

[46] Thöny-Meyer L, Ritz D, Hennecke H. Cytochrome *c* biogenesis in bacteria: a possible pathway begins to emerge. Molecular Microbiology 1994;12: 1–9. 8057830 10.1111/j.1365-2958.1994.tb00988.x

[47] Curson ARJ, Todd JD, Sullivan MJ, Johnston AWB. Catabolism of dimethylsulphoniopropionate: microorganisms, enzymes and genes. Nature Rev Microbiol 2011;9: 849–859. 10.1038/nrmicro2653 21986900

[48] Luo R, Liu B, Xie Y, Li Z, Huang W, Yuan J, et al SOAPdenovo2: an empirically improved memory-efficient short-read de novo assembler. Gigascience 2012;1: 18 10.1186/2047-217X-1-18 23587118 PMC3626529

[49] Zerbino DR, Birney E. Velvet: algorithms for de novo short read assembly using de Bruijn graphs. Genome Research 2008;18: 821–882. 10.1101/gr.074492.107 18349386 PMC2336801

[50] Bankevich A, Nurk S, Antipov D, Gurevich AA, Dvorkin M, Kulikov AS, et al (2012) SPAdes: a new genome assembly algorithm and its applications to single-cell sequencing. J Comput Biol 2012;19: 455–477. 10.1089/cmb.2012.0021 22506599 PMC3342519

[51] Gurevich A, Saveliev V, Vyahhi N, Tesler G. QUAST: quality assessment tool for genome assemblies. Bioinformatics 2013;29: 1072–1075. 10.1093/bioinformatics/btt086 23422339 PMC3624806

[52] Darling AE, Mau B, Perna NT. ProgressiveMauve: multiple genome alignment with gene gain, loss and rearrangement. PLoS One 2010;5: e11147 10.1371/journal.pone.0011147 20593022 PMC2892488

[53] Boetzer M, Henkel CV, Jansen HJ, Butler D, Pirovano W. Scaffolding pre-assembled contigs using SSPACE. Bioinformatics 2011;27: 578–579. 10.1093/bioinformatics/btq683 21149342

[54] Boetzer M, Pirovano W. Toward almost closed genomes with GapFiller. GenomeBiol 2012;13: R56 10.1186/gb-2012-13-6-r56 PMC3446322 22731987

[55] Altschul SF, Madden TL, Schäffer AA, Zhang J, Zhang Z, Miller W, et al Gapped BLAST and PSI-BLAST: a new generation of protein database search programs. Nucleic Acids Res 1997;25: 3389–3402. 9254694 10.1093/nar/25.17.3389 PMC146917

[56] Lu G, Moriyama EN. Vector NTI, a balanced all-in-one sequence analysis suite. Brief Bioinform 2004;5: 378–388. 15606974 10.1093/bib/5.4.378

[57] Petersen TN, Brunak S, von Heijne G, Nielsen H. SignalP 4.0: discriminating signal peptides from transmembrane regions. Nature Methods 2011;8: 785–786. 10.1038/nmeth.1701 21959131

[58] Edgar RC. MUSCLE: multiple sequence alignment with high accuracy and high throughput. Nucleic Acids Res 2004;32: 1792–1797. 15034147 10.1093/nar/gkh340 PMC390337

[59] Ronquist F, Teslenko M, van der Mark P, Ayres DL, Darling A, Höhna S, et al MrBayes 3.2: efficient Bayesian phylogenetic inference and model choice across a large model space. Syst Biol 2012;61: 539–542. 10.1093/sysbio/sys029 22357727 PMC3329765

